# Frequent *CLDN18-ARHGAP* fusion in highly metastatic diffuse-type gastric cancer with relatively early onset

**DOI:** 10.18632/oncotarget.25464

**Published:** 2018-06-29

**Authors:** Atsushi Tanaka, Shumpei Ishikawa, Tetsuo Ushiku, Sho Yamazawa, Hiroto Katoh, Akimasa Hayashi, Akiko Kunita, Masashi Fukayama

**Affiliations:** ^1^ Department of Pathology, Graduate School of Medicine, The University of Tokyo, Tokyo, Japan; ^2^ Department of Genomic Pathology, Medical Research Institute, Tokyo Medical and Dental University, Tokyo, Japan

**Keywords:** diffuse-type gastric cancer, CLDN18-ARHGAP fusion, E-cadherin, RHOA, CA9

## Abstract

CLDN18-ARHGAP26/6 fusions have been identified in gastric cancers, with a predominance in diffuse-type gastric cancers (DGCs). Although *in vitro* experiments have suggested an oncogenic role for *CLDN18-ARHGAP26/6* fusions, the exact frequencies and clinicopathological characteristics of the fusion-positive cases are poorly understood. We analyzed 254 cases of gastric cancer (172 diffuse-type and 82 intestinal-type) using RT-PCR and FISH, and also analyzed TCGA transcriptome datasets to identify genes that are related to the aggressive behaviors of fusion-positive cancers. Our assays identified 26 fusion-positive cases, 22 of which were DGCs (22/172, 12.8%). Unlike fusion-negative DGCs, almost all fusion-positive DGCs retained E-cadherin expression (P = 0.036). Fusion-positive DGCs also showed a higher prevalence of lymphatic and distant organ metastases, and these trends were only significant in the younger age group (< 60 years). In this group, the majority of cases with distant organ metastases (4 of 6 cases) were fusion-positive, and the multivariate regression analysis revealed that fusion status was an independent predictive marker for distant organ metastases (P = 0.002). In the TCGA dataset analysis, carbonic anhydrase 9 was postulated to be a potential modulator of the age-specific effects of the fusion protein, compatible with the immunohistochemical analysis of our cohort. Therefore, CLDN18-ARHGAP26/6 fusion-positive DGCs are considered biologically distinct entities that will require more advanced therapeutic options.

## INTRODUCTION

Diffuse-type gastric cancer (DGC) constitutes one of two major histological subtypes of gastric cancers, the other being intestinal-type gastric cancer (IGC). Histologically, DGC consists of poorly cohesive cancer cells with little or no glandular formation, frequently showing scirrhous growth patterns. Patients with DGC show poorer prognoses than those with IGC [1]. Whole-genome and whole-exome sequencing (WGS/WES) studies have shown that DGC rarely possesses amplifications of targetable receptor tyrosine kinases such as HER2, while recurrent *RHOA* mutations specifically occur in 14-25% of DGCs [2–4]. In addition, recurrent *CLDN18-ARHGAP* fusions have been identified predominantly among DGCs [4, 5], and, importantly, *RHOA* mutations and *CLDN18-ARHGAP* fusions were found to be mutually exclusive [4]. The frequencies of these fusions among gastric cancers has only been described in two cohorts and has been reported to be 14.8% among genomically stable (GS)-type gastric cancers in a study conducted by The Cancer Genome Atlas (TCGA) group [4], and 3.0% among 100 gastric cancers analyzed by Yao et al [5]. Therefore, the exact fusion frequency remains unclear and needs to be examined in an independent large cohort of gastric cancers.

**Figure 1:**
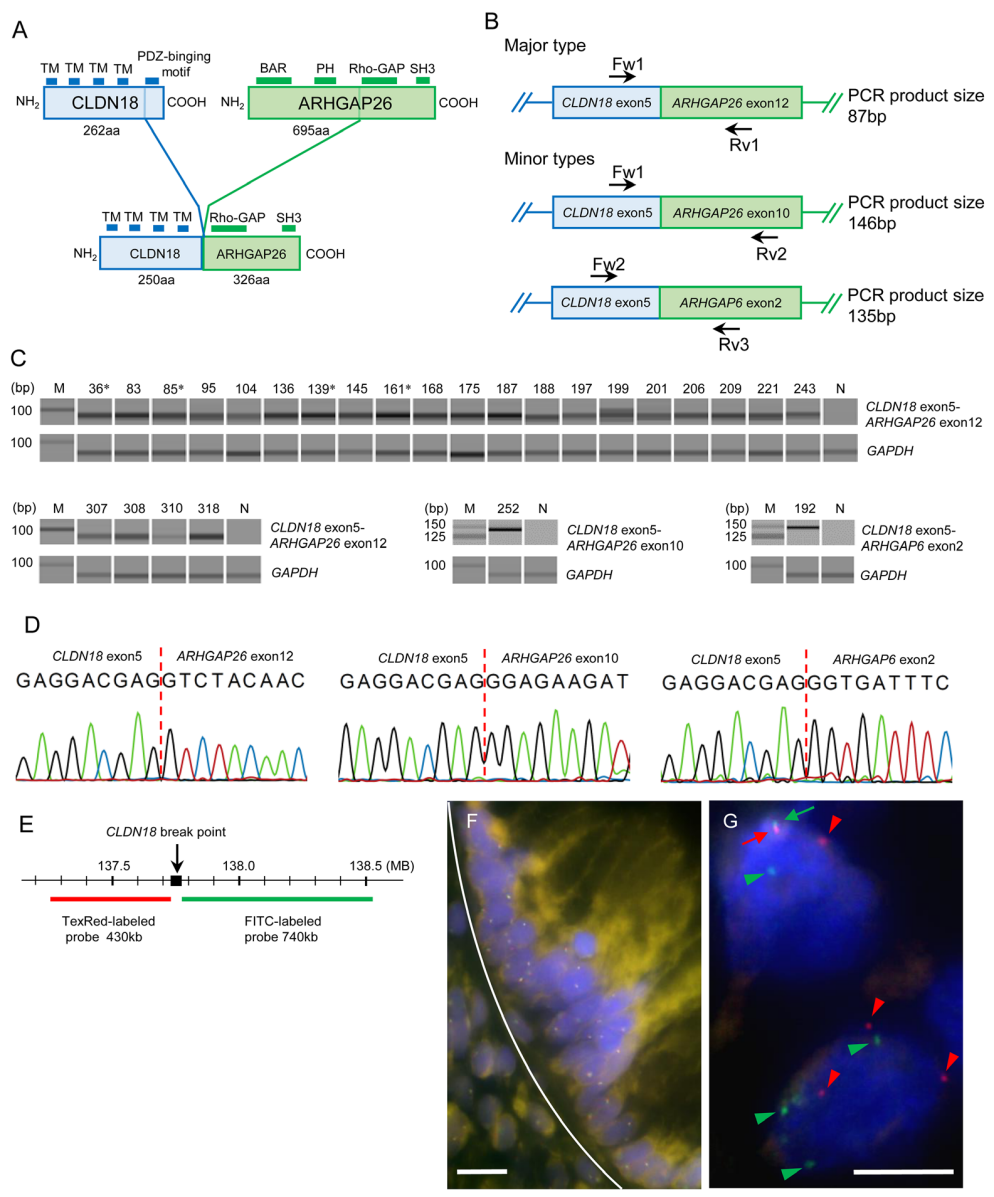
**(A)** Schematic diagram of the domains of wild-type CLDN18, wild-type ARHGAP26, and the major *CLDN18-ARHGAP26* fusion protein. The transmembrane domains (TMs) of CLDN18 and a RhoGAP domain of ARHGAP26 are retained in the fusion protein. The minor types of fusion proteins also have TMs and RhoGAP domains (not shown). **(B)** Schematic diagram of known *CLDN18-ARHGAP26/6* fusion transcripts. Primers used for these transcripts are indicated by arrows. **(C)** Fusion-positive RT-PCR results of 254 gastric cancer cases. Gel-like views of the electrophoretic assay are shown only for positive samples. There were 24 cases with *CLDN18-ARHGAP26* exon 12 (87bp), one with *CLDN18-ARHGAP26* exon 10 (146bp), and one with *CLDN18-ARHGAP6* exon 2 (135bp) fusion transcripts. The number above each band is the sample ID; M, molecular marker; N, non-tumor tissue template; ^*^, intestinal-type gastric cancer cases. **(D)** Sanger sequencing results of purified PCR products. Results of sample ID 83, 252, and 192 are shown. All amplicons were sequenced and confirmed to be fusion transcripts, all of which were in-frame and had no intercalating sequences. **(E)** Loci of FISH probes in CLDN18. Two DNA probes, labeled with Texas-red and FITC, respectively, were designed to hybridize upstream and downstream of CLDN18 break points. **(F)** A representative “no split” FISH signal of normal gastric mucosa. Upper side of white curved line represents the border of the normal epithelial gland layer. White bars: 5 μm. **(G)** Representative results of break-apart FISH signals for *CLDN18* in cancer cells (sample ID 187). Fused pair signals (red and green arrows) at the upper left side indicate un-rearranged normal *CLDN18*. Separated red signals (red arrow heads) and green signals (green arrow heads) indicate genomic rearrangement of *CLDN18*. White bars: 5 μm.

The *CLDN18-ARHGAP26/6* fusion gene retains the sequences of four transmembrane domains of CLDN18 and a RhoGAP domain of ARHGAP (Figure 1A); therefore, the protein encoded by the fusion gene is predicted to exert the RhoGAP activity of RHOA [6–8]. In addition, the fusion gene loses the cytoplasmic portion of CLDN18, which is involved in cell-cell adhesion through interactions with tight junction components [9–14]. In agreement, *in vitro* studies have demonstrated that *CLDN18-ARHGAP26*-transfected cancer cells showed reduced cell-cell adhesion and augmented invasiveness [5]. Considering these observations, it has been postulated that *CLDN18-ARHGAP26/6* fusions significantly impact the clinical behavior of gastric cancers. However, no studies to date have investigated the detailed clinicopathological features of fusion-positive gastric cancers.

In the present study, we examined 254 gastric cancer cases (172 diffuse-type and 82 intestinal-type) using several molecular pathology techniques combined with global gene expression analyses of public datasets, to determine the exact frequencies of the fusions and clarify the clinicopathological features of the fusion-positive cases. Our results revealed that fusion-positive DGCs tend to retain E-cadherin integrity and have a strong tendency towards advanced lymphatic and distant metastases among patients of younger ages. This study also suggests that the carbonic anhydrase 9 (CA9) enzyme is a possible modulator of age-specific fusion effects.

## RESULTS

### *CLDN18*-*ARHGAP26/6* fusion in gastric cancers analyzed by RT-PCR and FISH

The fusion transcripts (Figure 1A, 1B) were detected by RT-PCR in 22 out of the 172 DGC cases and four out of the 72 IGC cases (Figure 1C). Fusion types in DGCs were *CLDN18* exon 5-*ARHGAP26* exon 12 (n = 20), *CLDN18* exon 5-*ARHGAP26* exon 10 (n = 1), and *CLDN18* exon 5-*ARHGAP6* exon 2 (n = 1). Only the *CLDN18* exon 5-*ARHGAP26* exon 12 (n = 4) fusion was found in the IGC cohort. The sequences of all amplicons were confirmed by the Sanger method (Figure 1D, Supplementary Figure 1). In the FISH analysis (Figure 1G, Supplementary Figure 2), split signals of the *CLDN18* gene were identified in all RT-PCR positive gastric cancers. The mean percentage of cancer cells that showed split signals in each case was 16.4 ± 8.8% (average ± standard deviation), ranging from 9.2% to 48.0%.

### Immunohistochemical analysis of CLDN18 and ARHGAP26

**Figure 2:**
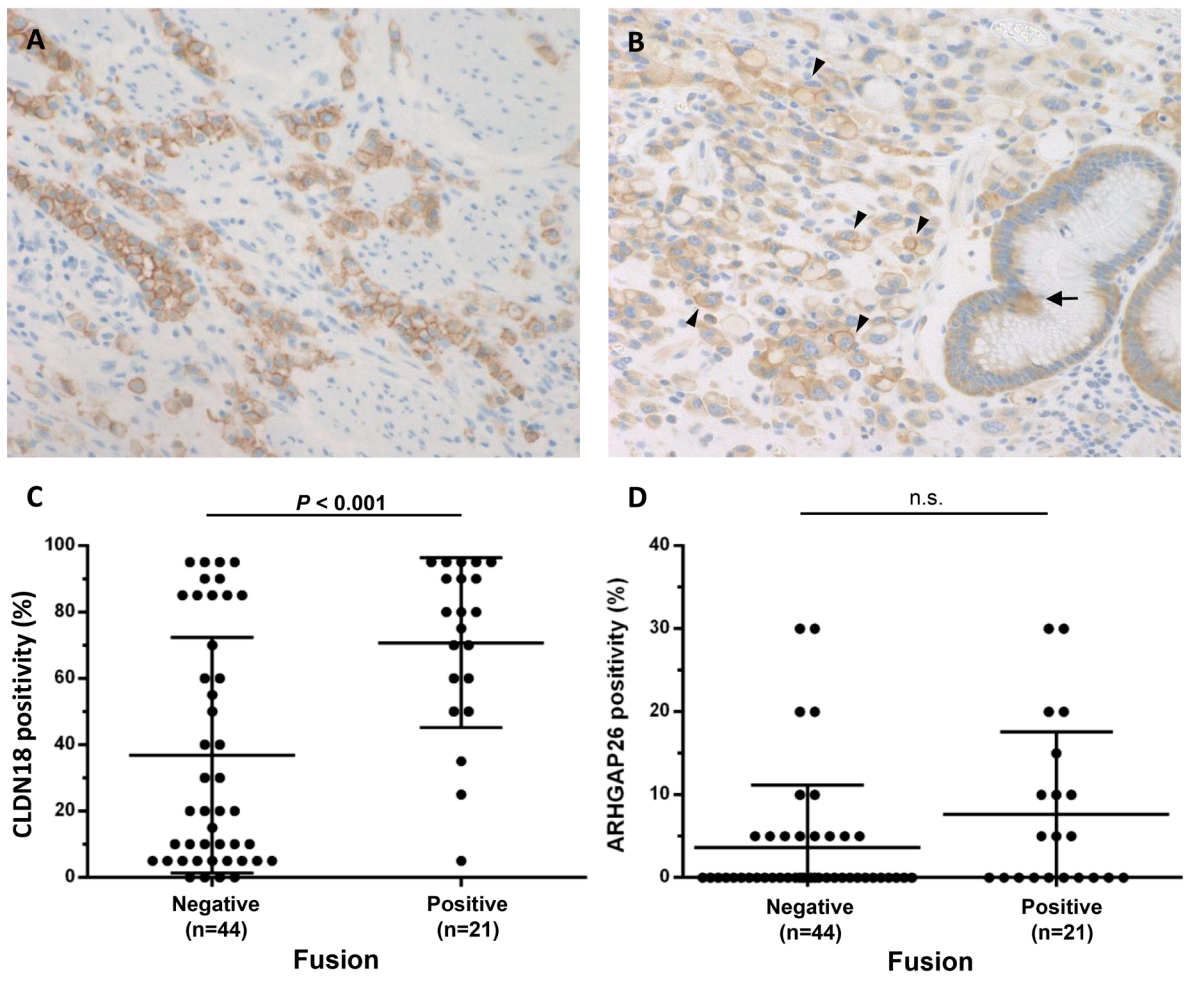
CLDN18 and ARHGAP26 expression in DGCs with fusions **(A)** Representative results of CLDN18 immunohistochemistry showing diffuse protein expression in tumor cells. Both partially and entire circumferential membranous expression were observed (magnification 200x). **(B)** Representative ARHGAP26 immunohistochemistry results showing cytoplasmic and membranous expression of ARHGAP26 in normal (arrow) and cancer (arrow head) cells (magnification 200x). **(C, D)** Each figure shows expression levels of CLDN18 (C) and ARHGAP26 (D) in cancer cells, evaluated by immunohistochemistry. For both CLDN18 and ARHGAP26, only membranous staining was counted as positive. Almost all of the fusion-positive cancers diffusely expressed CLDN18, whereas fusion-negative cancers showed low CLDN18 expression (*P* = 0.0001). Membranous immunostaining of ARHGAP26 is observed in 11 of 21 (52.4%) fusion-positive cases and in 14 of 44 (31.8%) fusion-negative cases (*P* = 0.172). Numbers under each plot indicate the sample sizes of each group.

To evaluate the correlations between gene fusions and protein expression of CLDN18 and ARHGAP26, immunohistochemical analyses were performed for cases of DGC that were either with or without *CLDN18*-*ARHGAP26* fusions (n = 21 and 44, respectively). CLDN18 immunostaining was observed in cancer cells at the apical and lateral membranes of glandular cells and occasionally on the entire circumference of infiltrating cells (Figure 2A). Diffuse CLDN18 immunostaining (> 50%) was observed in 18 of 21 fusion-positive cases, but only in 15 of 44 fusion-negative cases (*P* = 0.0001). Almost all fusion-positive cancers showed positive CLDN18 immunostaining, suggesting that CLDN18-ARHGAP fusion expression is indispensable in the cases with gene fusions. Cytoplasmic ARHGAP26 immunostaining was observed in both normal and cancer cells (Figure 2B). In addition, coexisting membranous immunostaining was observed in 11 of 21 (52.4%) fusion-positive cases, but in 14 of 44 (31.8%) fusion-negative cases (*P* = 0.172). Although DGCs with fusions tended to show membranous staining of ARHGAP26, there were no statistically significant differences in the staining frequencies from fusion-negative DGCs.

### Clinicopathological analysis of DGCs with and without *CLDN18-ARHGAP26/6* fusions

**Figure 3:**
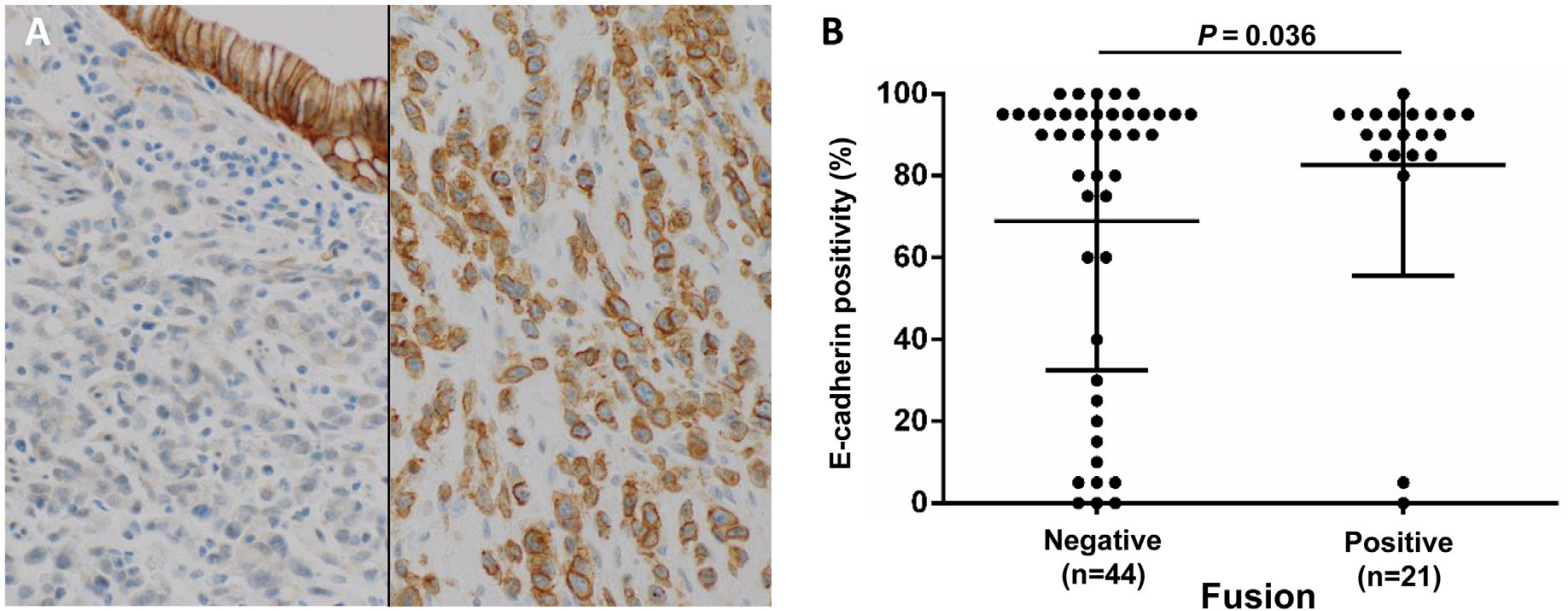
Diffuse E-cadherin expression (80% or more positivity of tumor cells) correlates with fusion status **(A)** Representative E-cadherin immunohistochemistry showing diffuse positive staining in a fusion-positive case (right) and negative staining in a fusion-negative case, with positive staining in normal epithelial cells (left) (magnification 200x). **(B)** E-cadherin-positive cell fractions in each DGC case, among fusion-positive and fusion-negative cases. Unlike fusion-negative cases, most fusion-positive cases retained E-cadherin expression (*P* = 0.036). Fisher's exact test was performed between the high expression group (80% positivity or more) and low expression group. Numbers under each plot indicate the sample sizes of each group.

The clinicopathological features of DGCs with and without CLDN18-ARHGAP26/6 fusions are presented in Table 1a. Fusion-positive DGCs showed significantly larger sizes (*P* = 0.013, mean tumor size: 8.5 vs. 6.3 cm), and lymph node and distant organ metastases were more frequently observed in fusion-positive DGCs than in fusion-negative DGCs (*P* = 0.014 and 0.003, respectively). For fusion-positive cases, organ metastases were detected in the liver (2 cases); pancreas and retroperitoneum (1 case); and liver, pancreas, and ovaries (1 case). For fusion-negative cases, tumor metastases were detected in the ovary (1 case) and liver (1 case). As loss of E-cadherin expression is one of the most well-known aberrations in DGC, we examined E-cadherin immunohistochemical staining in our DGC cohort. Interestingly, while a portion of the fusion-negative DGCs (16/28, 36.3%) showed loss of E-cadherin immunoreactivity, most of the fusion-positive DGCs (19/21, 90.4%) retained positive E-cadherin staining (*P* = 0.036) (Figure 3A, 3B).

**Table 1A:** Clinicopathological characteristics of DGC with the fusions

Feature	Diffuse-type GC (n = 172)		*P*-value
	Fusion negative (n = 150)	Fusion positive (n = 22)	
Gender			1.000
Men	91	13	
Women	59	9	
Mean age and range (years)	60 (31-87)	59 (33-76)	0.755^a^
Locus			0.529
Proximal third	23	5	
Middle third	76	9	
Distal third	51	8	
Mean tumor size and range (cm)	6.3 (0.8-20)	8.5 (2.5-20)	*0.013*^a^
Macroscopic type			
Early cancer (n = 75)			1.000
Superficially depressed type	67	8	
Advanced cancer (n = 97)			0.545
Bormann type 2	5	0	
Bormann type 3	48	7	
Bormann type 4	30	7	
Lymphatic invasion			0.108
Absent	90	9	
Present	60	13	
Venous invasion			0.070
Absent	80	7	
Present	70	15	
T stage			0.117
T1-2	82	8	
T3-4	68	14	
N stage			*0.014*
N0-1	109	10	
N2-3	41	12	
M stage			
Distant organ metastasis			*0.003*
Absent	148	18	
Present	2	4	
Peritoneal dissemination or malignant ascites			0.517
Absent	130	18	
Present	20	4	
Extraregional lymphnode metastasis			1.000
Absent	144	21	
Present	6	1	
Stage			0.095
I + II	99	10	
III + IV	51	12	

^a^, Mann–Whitney *U* test

**Table 1B:** Clinicopathological characteristics of DGCs with the fusions among patients under 60-year-old

Feature	Diffuse-type GC (n = 87)		*P*-value
	Fusion negative (n = 76)	Fusion positive (n = 11)	
Gender			0.750
Men	46	6	
Women	30	5	
Mean age and range (years)	51 (31-59)	48 (33-59)	0.293^a^
Locus			0.543
Proximal third	10	2	
Middle third	42	4	
Distal third	24	5	
Mean tumor size and range (cm)	6.5 (0.8-20)	7.9 (2.5-15)	0.143^a^
Macroscopic type			
Early cancer (n = 75)			1.000
Superficially depressed type	34	2	
Advanced cancer (n = 97)			1.000
Bormann type 2	3	0	
Bormann type 3	22	5	
Bormann type 4	17	4	
Lymphatic invasion			*0.018*
Absent	51	3	
Present	25	8	
Venous invasion			0.105
Absent	43	3	
Present	33	8	
T stage			*0.021*
T1-2	44	2	
T3-4	32	9	
N stage			*< 0.001*
N0-1	60	2	
N2-3	16	9	
M stage			
Distant organ metastasis			*0.002*
Absent	74	7	
Present	2	4	
Peritoneal dissemination or malignant ascites			0.374
Absent	65	8	
Present	11	3	
Extraregional lymphnode metastasis			0.424
Absent	73	10	
Present	3	1	
Stage			*0.002*
I + II	52	2	
III + IV	24	9	

^a^, Mann–Whitney *U* test.

**Table 1C:** Clinicopathological characteristics of DGCs with the fusions among older patients (≥ 60 years)

Feature	Diffuse-type GC (n = 85)		*P*-value
	Fusion negative (n = 74)	Fusion positive (n = 11)	
Gender			1.000
Men	45	7	
Women	29	4	
Mean age and range (years)	70 (60-87)	70 (63-76)	0.969^a^
Locus			0.642
Proximal third	13	3	
Middle third	34	5	
Distal third	27	3	
Mean tumor size and range (cm)	6.1 (1.2-19.5)	9.0 (3.3-20)	*0.036*^a^
Macroscopic type			
Early cancer (n = 75)			1.000
Superficially depressed type	33	6	
Advanced cancer (n = 97)			0.474
Bormann type 2	2	0	
Bormann type 3	26	2	
Bormann type 4	13	3	
Lymphatic invasion			1.000
Absent	39	6	
Present	35	5	
Venous invasion			0.523
Absent	37	4	
Present	37	7	
T stage			1.000
T1-2	38	6	
T3-4	36	5	
N stage			1.000
N0-1	49	8	
N2-3	25	3	
M stage			1.000
Distant organ metastasis			1.000
Absent	74	11	
Present	0	0	
Peritoneal dissemination or malignant ascites			1.000
Absent	65	10	
Present	9	1	
Extraregional lymphnode metastasis			1.000
Absent	71	11	
Present	3	0	
Stage			1.000
I + II	47	8	
III + IV	27	3	

^a^, Mann–Whitney *U* test

Distant metastases were observed among relatively young patients who were less than 60 years of age. Therefore, the clinicopathological factors of the DGCs with or without gene fusions were further examined statistically between younger patients and those older than 60 years (Table 1b, 1c). In the older age group, there were no statisticaly significant differences in the examined clinicopathological factors, with the exception of a mild difference in the mean tumor sizes of DGCs with and without fusions. On the other hand, in the younger age group, fusion-positive DGCs showed higher T factors, higher N factors, and more frequent distant organ metastases (Table 1b). In our cohort, while distant organ metastases were not observed in the older age group, most (4 out of 6) of the cases with distant organ metastases in the younger age group were fusion-positive. The multivariate analysis of younger patients with DGCs revealed that fusion status and peritoneal dissemination/malignant ascites statuses were independent predictive factors of distant organ metastasis (Table 2).

**Table 2:** Univariate and multivariate analyses of distant organ metastases in DGCs in younger individuals

Features	Distant organ metastasis		Univariate	Multivariate	
	Absent	Present	*P*-value	Odds ratio (95% C.I.)	*P*-value
Gender			*0.037*	-	-
Men	51	1			
Women	30	5			
Locus			1.000	-	*-*
Proximal third and middle third	54	4			
Distal third	27	2			
Tumor size (cm)			*0.027*	-	*-*
< 5.0	42	0			
≥ 5.0	39	6			
Lymphatic invasion			*0.028*	-	*-*
Absent	53	1			
Present	28	5			
Venous invasion			*0.009*	-	-
Absent	46	0			
Present	35	6			
Fusion			*0.002*		*0.002*
Absent	74	2		Reference	
Present	7	4		30.657 (3.570-680.338)	
T stage			*0.009*	-	-
T1-2	46	0			
T3-4	35	6			
N stage			*0.007*	-	-
N0-1	61	1			
N2-3	20	5			
Peritoneal dissemination or malignant ascites			*0.006*		*0.005*
Absent	71	2		Reference	
Present	10	4		20.965 (2.436-465.157)	
Extraregional lymph node metastasis			0.253	-	-
Absent	78	5			
Present	3	1			

### Age-related changes in genomic status and gene expression profile

In the present study, influences of fusion genes on malignant phenotypes were observed only in the younger age group. Therefore, we tested whether differences existed between younger and older age groups, in background mutation densities of whole cancer exomes and/or frequencies of 25 driver gene mutations (*TP53*, *CDH1*, *SMAD4*, *PIK3CA*, *RHOA*, *ARID1A*, *KRAS*, *MUC6*, *APC*, *BCOR*, *EYA4*, *BNC2*, *RNF43*, *ABCA10*, *CTNNB1*, *MACF1*, *SMAD2*, *SOHLH2*, *RASA1*, *FAM46D*, *PLB1*, *CNGA4*, *AGO4*, *ERBB2*, *PTPRC*, which were reported as significantly mutated genes in non-hypermutated gastric cancers in the TCGA study). Significant correlations were not observed between patient age and whole exome mutation density or driver gene mutation frequency (data not shown).

**Figure 4:**
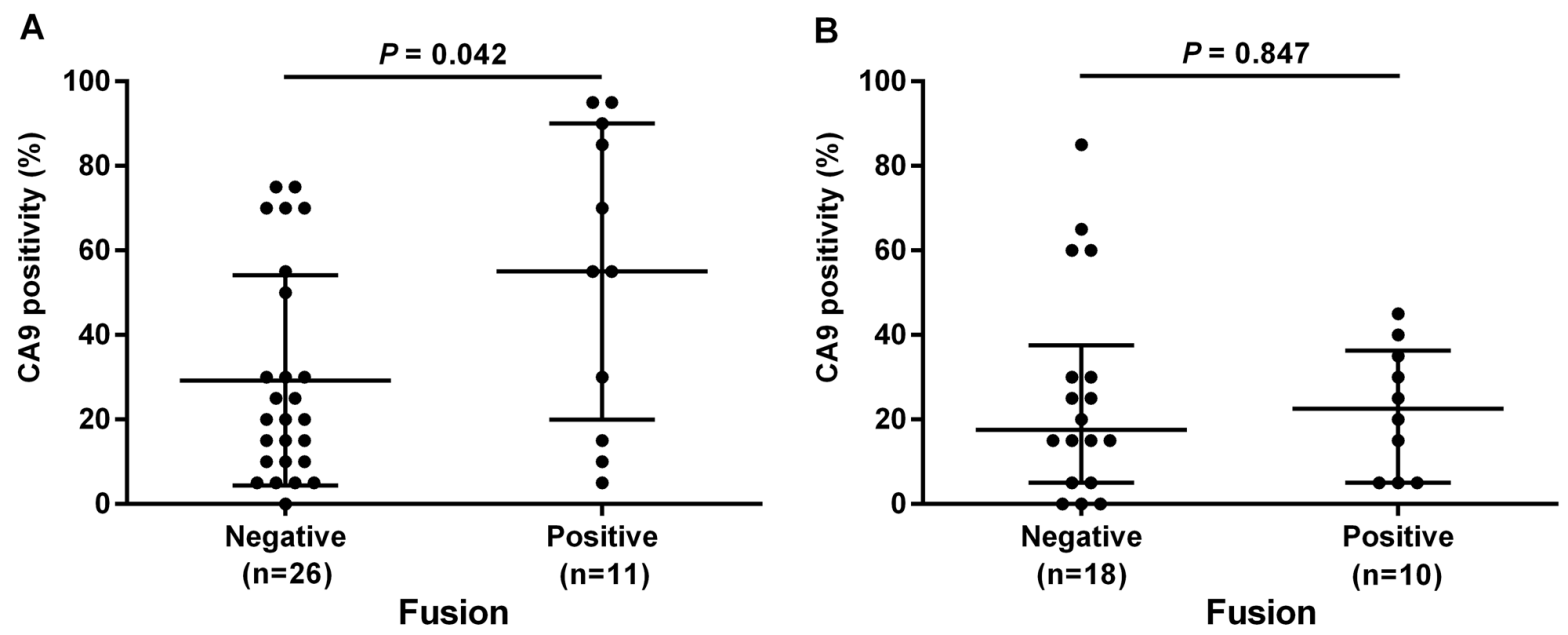
Relationships between CA9 expression and *CLDN18-ARHGAP26* fusion status evaluated by immunohistochemistry Younger age patients (under 60 years) **(A)**, and older age patients (older than 60 years) **(B)** were evaluated separately. Fusion-positive DGC cases showed significantly higher CA9 expression than fusion-negative cases, only in the younger age group (*P* = 0.042). Numbers under each plot indicate the sample sizes of each group.

To obtain molecular insights regarding the age-specific fusion effects, we examined differences in gene expression profiles between younger and older individuals with DGCs with fusions (13 TCGA cases in total). We selected up- or down-regulated genes, with RNA expression levels that significantly changed by more than 5-fold between younger and older groups with the fusions. We identified 11 upregulated and 22 downregulated genes in the younger group (Supplementary Table 1). Among these genes, we focused on CA9, because previous studies have shown that CA9 promotes tumor cell migration and invasion *in vitro* by inhibiting the RHOA pathway, and is highly expressed in younger patients with cervical and hepatocellular malignancies [15, 16]. As stated above, CLDN18 fusions are expected to inhibit the RHOA pathway [5], and in some contexts, RHOA inhibition promotes cancer cell invasion *in vitro* [17]. Based on these observations, we hypothesized that the malignant phenotypes of cancer cells are further exaggerated by CA9 expression in younger individuals with DGC. To validate the relationship of CLDN18 fusions with age and CA9 expression level, we performed immunohistochemical analyses of CA9 across 65 selected DGCs, as described in the Materials and Methods section (Figure 4). In agreement with the results from the TCGA dataset, CA9 immunohistochemistry showed higher protein expression in the younger group than in the older group (*P* = 0.152). In the younger group, CA9 expression was significantly higher in fusion-positive cancers than in fusion-negative cancers (*P* = 0.042). Moreover, CA9 expression was higher in cases with distant organ metastases (*P* = 0.059). On the other hand, in the older group, no statistical associations were observed between the CA9 expression and fusion status.

## DISCUSSION

In the present study, 26 of the 254 gastric cancers examined in our cohort had *CLDN18-ARHGAP26/6* fusions, and 22 of the 26 gastric cancers with the fusions were DGCs (22 cancers of 172 DGCs, 12.8%). The frequency of the CLDN18-ARHGAP26/6 fusions among the DGCs was consistent with the TCGA data, from which eight (14.8%) of 54 GS-type cancers had the fusions [4]. The frequency observed in our study is also considered comparable with the frequency that was reported in the study of Yao et al., in which three cases were fusion-positive among 100 gastric cancers, including 30 DGC cases [5].

Our clinicopathological analyses showed that gastric cancers with the fusions tended to have larger diameter and more lymph node and distant metastases than gastric cancers without the fusions. Most (4 out of 6) of the gastric cancers with distant organ metastases were *CLDN18-ARHGAP26/6* fusion-positive. We also observed that fusion status and peritoneal dissemination/malignant ascites were independent predictive factors for distant organ metastases in younger individuals with DGC. Therefore, fusion status is a clinically important and useful biomarker, in consideration of therapeutic strategies. Although our study is the largest study to have focused on DGCs with *CLND18* fusions [4, 5], only 22 of the included patients had fusion-positive DGCs. Therefore, more patients with fusion-positive DGCs should be further examined, to obtain definitive conclusions.

CLDN18 interacts with tight junction proteins via a PDZ-domain binding motif in its C-terminus and maintains cell-cell or cell-extracellular matrix adhesions [18, 19]. Because CLDN18-ARHGAP26/6 lacks the cytoplasmic portion (C-terminus region) of CLDN18 and the N-terminal domain of ARHGAP26/6 (Figure 1A), it is possible that the fusion protein alters cell-cell or cell-extracellular matrix adhesions. In addition, aberrant regulations of the Rho pathway, which could take place upon fusion of ARHGAP with a RhoGAP domain to CLDN18 with transmembrane domains, may influence tumor cell movements and invasiveness. In agreement, an *in vitro* analysis that was reported by Yao et al. demonstrated that tumor cells stably expressing CLDN18-ARHGAP26 showed weak cell-cell adhesions and strong invasiveness, through inhibition of the RHOA pathway [5]. Furthermore, E-cadherin expression is preserved, with significantly higher rates in fusion-positive DGCs than in fusion-negative DGCs. Consistent with these findings, the TCGA data also showed that the *CLDN18-ARHGAP26/6* fusion was mutually exclusive with *CDH1* mutations. Taking the above-mentioned results into consideration, the *CLDN18-ARHGAP26/6* fusion is speculated to be a strong driver that substantially influence both abnormal RHOA signaling and cell-cell adhesions, which could otherwise be achieved by*RHOA* and *CDH1* mutations. This hypothesis is consistent with the highly aggressive clinicopathological features of gastric cancers with the fusions, as shown in the present study.

Our detailed clinicopathological analyses of fusion-positive cases revealed that the fusion status had strong impact on tumor progressions, i.e., higher T, N, and M stages, only in the younger age group, and such correlations were not observed in the older age group. We then focused on the relationship between gene fusion and CA9 expression and found, in our cohort, that CA9 expression levels were higher in fusion-positive cases only among the younger age group. In addition to the evidence for correlations between CA9 and RHOA functions [16, 17], CA9 has also been found to promote cervical cancer cell invasions by inhibiting RHOA *in vitro* [20]. These results suggest that, in younger patients, fusion-positive gastric cancer cells invade and metastasize through fusion protein-mediated inhibition of RHOA and elevated expression of CA9.

Our immunohistochemical analysis showed that almost all *CLDN18-ARHGAP26* fusion-positive cases expressed CLDN18, about half of which expressed ARHGAP26 in the membrane, which likely reflects the requirement of *CLDN18* promoter activity for expression of the fusion protein in tumor cells. On the other hand, ARHGAP26 is usually expressed in the cytoplasm of normal tissue cells and some tumor cells [21]. However, we detected membranous expression of ARHGAP26 in about half of the fusion-positive cases and about one-quarter of the fusion-negative cases. The higher rate of membranous staining of ARHGAP26 in the fusion-positive cases can be explained by the structure of the N-terminal membrane protein CLDN18 in the fusion. The lack of membranous staining of ARHGAP26 in some of the fusion-positive cases might be due to the problems with detection sensitivity, for example, due to low expression of the fusion protein or conformation changes of the antibody-targeted C-terminus region, which could be caused by N-terminal fusion. It remains unclear why some of the fusion-negative cases also showed membranous staining patterns. One possible reason involves the interaction with membrane-localized focal adhesion kinase (FAK), via the SH3 domain [6, 22]. FAK is known to regulate cell adhesion, motility, proliferation, and survival in many cell types [23] and is overexpressed in various cancers, including gastric cancer [4, 24]. This partially explains the membranous staining of ARHGAP26. Although the relationship between fusion status and immunolocalization was not so completely parallel, the driver nature of the fusion has been clearly shown by Yao's report [5]. They showed that the fusion induced the epithelial mesenchymal transition and increased cancer invasiveness by inhibiting the RHOA pathway. In the TCGA datasets, the fusion and *RHOA* mutations were mutually exclusive. This mutual exclusivity was confirmed in our study (data not shown) and provides strong genetic evidence that both the *CLDN18-ARHGAP* fusion and *RHOA* mutations are cancer drivers in the same pathway. In this context, additional RHOA pathway inhibition is induced by the CA9 protein and, as is discussed above, deterioration of cell-cell adhesion by the fusion protein is responsible for the distinctly aggressive malignant feature of fusion-positive cancers.

In summary, we investigated the clinicopathological characteristics of *CLDN18-ARHGAP26/6* fusion-positive gastric cancers and found that fusion-positive DGCs have strong metastatic ability, a phenotype that is more obvious in younger patients. We identified CA9, which is highly expressed in fusion-positive cases of younger individuals, as a potential modulator of abnormal RHOA signaling and cell adhesion by the fusion protein. As is suggested by the very low frequency of E-cadherin aberrations among fusion-positive DGCs, this type of gastric cancer could have different biological characteristics from usual DGCs. Therefore, CLDN18-ARHGAP26/6 fusion is a clinically relevant biomarker for the prediction of highly aggressive DGCs, for which more advanced therapeutic options are necessary. To this point, the CLDN18-ARHGAP26/6 fusion protein, a driver that contributes to highly aggressive phenotypes, is a strong drug target candidate, based on its accessibility on the cell membrane and its complete absence in non-cancer cells. In fact, an anti-CLDN18 monoclonal antibody, claudiximab [25], has been developed for gastric cancers and is currently being evaluated in clinical trials. It is also possible that information regarding fusion status constitutes a useful biomarker for selecting patient subgroups with distinct responses.

## MATERIALS AND METHODS

### Case selection and clinical data

The pathological records of 1,163 patients with gastric cancers at the University of Tokyo Hospital from 2000 to 2013 were available and reviewed by two pathologists (A.T. and T.U.). To obtain a gastric cancer cohort of appropriate size, 172 consecutive cases of DGC were selected. Exclusion criteria were: (i) small tumor size (< 0.5 cm), (ii) Epstein-Barr Virus-associated gastric carcinomas; and (iii) cases used in the previous WES study [3]. Eighty-two cases of IGC were also selected as controls.

Clinical data including symptoms, laboratory data, endoscopic findings, and imaging data were extracted from medical records. Macroscopic tumor types were classified according to the criteria of the World Health Organization (WHO) classification for early gastric cancers and the Borrmann classification for advanced gastric cancers [26]. Tumor stages were determined using the 8th edition American Joint Committee on Cancer guideline for tumor, node, and metastasis (TNM) classification. All research protocols in the present study were approved by our institutional review boards.

### Histological assessment

Two pathologists reviewed hematoxylin and eosin-stained (H-E) sections of all gastric cancers in this study, and determined the lesion size, depth of invasion, vascular invasion, lymph node metastasis, Lauren's classification, and pathological stages according to the criteria of the WHO classification criteria [26].

### RNA extraction

Ten-micrometer-thick sections were sliced from the formalin-fixed paraffin embedded (FFPE) tissues and mounted onto glass slides. Adjacent 4-micrometer-thick sections were subjected to the H-E staining to confirm the tumor contents, after which the tumor areas were manually dissected from the corresponding 10-micrometer sections. RNA was extracted using the RecoverAll™ Total Nucleic Acid Isolation kit (Thermo Fisher Scientific, Waltham, MA, USA) according to the manufacturer's instructions.

### RT-PCR for *CLDN18-ARHGAP* transcripts

Total RNA was subjected to complementary DNA (cDNA) synthesis with random primers using PrimeScript™ RT reagent Kit with gDNA Eraser (Takara Co., Ltd., Kyoto, Japan). PCR reactions were then performed in a reaction volume of 10 μl containing 0.2 mM dNTP, 2 μl 5X PrimeSTAR GXL Buffer, 0.2 μl PrimeSTAR GXL DNA Polymerase (all from Takara), 0.3 μM forward primer, 0.3 μM reverse primer, and 1 μl cDNA to detect fusion transcripts (Figure 1B). PCR primers were as follows: *CLDN18* exon 5-*ARHGAP26* exon 12 (Fw1 primer: 5’-TTGGGTCCAACACCAAAAAC-3’, Rv1 primer: 5’-TCTGGCTGTCTTTGTTCGAG-3’, product size 87 bp), *CLDN18* exon 5-*ARHGAP26* exon 10 (Fw1, Rv2 primer: 5’-TGCTTCCACATCAAAGCAAA-3’, product size 146 bp) and *CLDN18* exon 5-*ARHGAP6* exon 2 (Fw2 primer: 5’-GCCACAGTGTTGCCTACAAG-3’, Rv3 primer: 5’-CTGACATGCTGTTCCAGGTG-3’, product size 135 bp). We also evaluated the cDNA qualities using primers for *GAPDH* (forward: 5’-CAACGGATTTGGTCGTATTGG-3’, reverse: 5’-GCAACAATATCCACTTTACCAGAGTTAA-3’, product size 72 bp). PCR thermal cycling conditions were as follows: initial denaturation at 98°C for 2 min, followed by 45 cycles of denaturation at 98°C for 20 s, annealing at 55°C for 15 s and extension at 68°C for 30 s, and final extension at 68°C for 3 min. The PCR product for each case was visualized using a capillary electrophoresis machine, MCE®-202 MultiNA (Shimadzu, Kyoto, Japan). All amplicons targeting fusion genes were sequenced by the Sanger method.

### Fluorescence *in situ* hybridization for *CLDN18* gene translocation

All cases that showed RT-PCR positivity were analyzed by fluorescence *in situ* hybridization (FISH), a dual-color *CLDN18*-split assay. Four-micrometer-thick sections were de-paraffinized in xylene, dehydrated in ethanol, and dried. The sections were then incubated with Pretreatment Solution (GSP Lab, Inc., Hyogo, Japan) at 95-100°C for 30 min, then treated with 0.8% pepsin solution (Sigma P-7125, Sigma-Aldrich Corp., St. Louis, MO, USA) in 0.2 N HCl at 37°C for 10 min. The sections were washed in 2 x saline-sodium citrate (SSC), then in ethanol, and air dried. Dual-color break-apart FISH for the detection of *CLDN18* gene translocations was performed by using commercially available probes (GSP Lab, Inc.). The first 430 kb long probe labelled with Texas Red recognized the proximal region of the *CLDN18* gene, whereas the second FITC-labelled probe corresponded to the 740 kb distal (3′) region of the *CLDN18* gene (Figure 1E). The probe set for *CLDN18* was applied to the sections according to the manufacturer's instructions and hybridized at 37°C for 48 hours. After hybridization, the sections were washed in 2 x SSC/0.3% Nonidet P40 (Sigma-Aldrich) at 72°C for 5 min and subsequently washed in ethanol twice. Then, the sections were counterstained with DAPI, 4’6-diamidino-2-phenylindole. The slides were visualized with the Leica LM6000B imaging system (Leica Microsystems, Wetzlar, Germany) and analyzed using LAS X version 1.0 imaging software (Leica Microsystems). Split signal was defined as signals observed 2 or more diameters apart. Samples were considered positive for *CLDN18* gene translocation when more than 5% of tumor cells harbored split signals, counting at least 100 cells. The mean split signal rate of the normal background epithelia in 5 gastric cancer cases was only 0.4% with a standard deviation of 0.54 (Figure 1F).

### Case selection for immunohistochemistry of CLDN18, ARHGAP26, CA9, and E-cadherin

To evaluate protein expression patterns of CLDN18, ARHGAP26, CA9, and E-cadherin in gastric cancers by immunohistochemistry, we selected 65 representative cases: 21 DGCs with the CLDN18-ARHGAP26 fusions and 44 DGCs without the fusions as controls. Control cases were matched in terms of locus, T stage, N stage, M stage, age, sex, tumor size, and lymphovascular invasion status.

### Immunohistochemistry

FFPE tissues were cut into 4-micrometer-thick sections and de-paraffinized with xylene, after which an antigen retrieval procedure was performed. Immunohistochemical analyses were conducted with antibodies against CLDN18 (Product No. 38-8000, 1:1000, Thermo Fisher Scientific), ARHGAP26 (HPA035107, 1:50, Atlas antibodies, Bromma, Sweden), CA9 (AB108351, 1:250, Abcam, Inc., Cambridge, CA, USA), and E-cadherin (Product No. 610182, 1:200, BD Biosciences, San Jose, CA, USA) using a Ventana Benchmark XT autostainer system (Ventana Medical Systems, Inc., Tucson, AZ, USA). Immunostaining was semi-quantitatively evaluated (0 to 100, with 5 % steps) according to the proportions of positive membrane staining among cancerous cell populations. Evaluation of immunohistochemistry was independently performed by two pathologists (A.T. and T.U.). In cases with discrepancies in the immunohistochemistry scores between the two pathologists, they reviewed the cases in together and consensus scores were determined.

### Extraction and analysis of TCGA data

Genomic status (e.g., driver mutations and mutation frequencies), RNA-sequencing data, and clinical information of TCGA stomach adenocarcinoma cases were downloaded from cBioPortal site (http://www.cbioportal.org/). We found 255 age-identified samples and classified the subjects into four subgroups according to fusion statuses and ages (< 60 or ≥ 60). We analyzed associations between numbers of somatic mutations among 25 driver genes, exome-wide mutation densities, and patient ages. Then, to find genes related to the age-specific fusion effects on aggressive behaviors of cancer cells, we selected up- or downregulated genes with RNA expression levels that significantly changed more than 5 times between young and old groups among fusion-positive DGCs.

### Statistical analysis

Patient ages and tumor sizes derived from the TCGA data were compared between groups using the Mann–Whitney *U* test. Other categorical variables were compared using Fisher's exact test. Multivariate analyses of distant organ metastases using factors which showed *P*-values < 0.05 in univariate analysis were performed with the logistic regression model. A backward elimination method was used with a threshold of *P* = 0.05 to select variables for the final model. Statistical analysis was performed using JMP Pro 11 software (SAS, Cary, NC, USA). RNA expression data were analyzed using CLC Main Workbench 7 software (QIAGEN Inc., Valencia, CA, USA) and the *t*-test. All statistical analyses were considered significant with *P*-values < 0.05.

## SUPPLEMENTARY MATERIALS FIGURES AND TABLES



## Abbreviations

DGC: Diffuse-type gastric cancer
IGC: Intestinal-type gastric cancer
CA9: carbonic anhydrase 9
WGS: Whole-genome sequencing
WES: whole-exome sequencing
GS: genomically stable
TCGA: The Cancer Genome Atlas Network
WHO: World Health Organization
TNM: tumor, node, and metastasis
FFPE: formalin-fixed paraffin embedded
cDNA: complementary DNA
FISH: fluorescence *in situ* hybridization
SSC: saline-sodium citrate
