# Supplementary material for: Frequent CLDN18-ARHGAP fusion in highly metastatic diffuse-type gastric cancer with relatively early onset
Source: Oncotarget. 2018 Jun 29;9(50):29336–50. doi: 10.18632/oncotarget.25464 (PMC6047683; doi:10.18632/oncotarget.25464)
Supplement: Supplementary file 1 [file oncotarget-09-29336-s001.pdf]

Frequent *CLDN18-ARHGAP* fusion in highly metastatic diffuse-type gastric cancer with relatively early onset

SUPPLEMENTARY MATERIALS

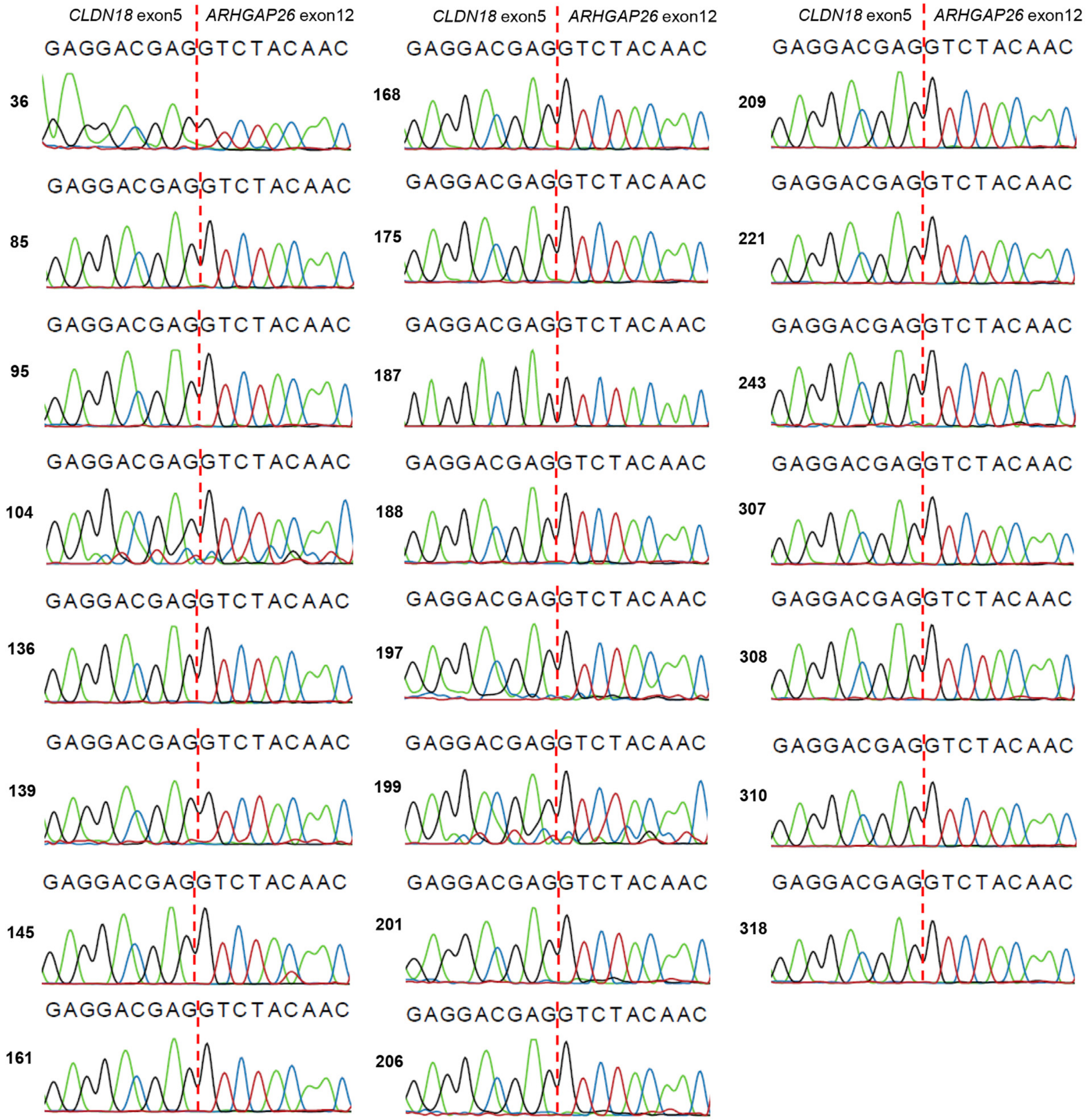

Supplementary Figure 1: Sanger sequencing results of purified PCR products are shown here. All amplicons were confirmed to be fusion transcripts. Sample ID are shown on the left of the tracing data.

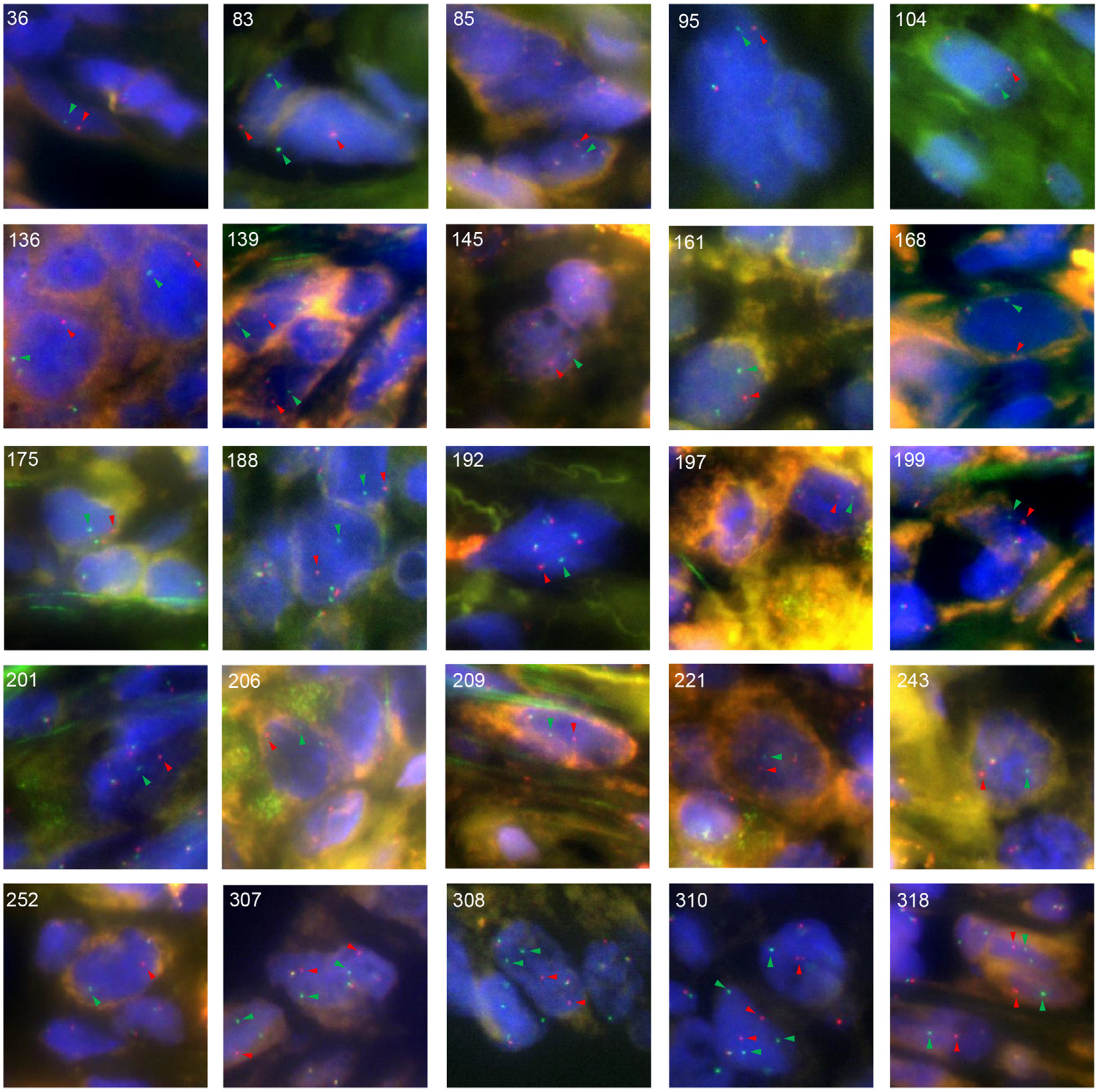

Supplementary Figure 2: FISH results of RT-PCR positive cases. All samples had split signals (red and green arrow heads). Sample ID are shown on the left of the each photo.

Supplementary Table 1: Differentially expressed genes in gastric cancers with *CLDN18* fusions in younger individuals

Genes	Fold change (v.s. old patients)	P-value
MIR33B	20.666	0.035
TCEB3B	18.339	0.026
BOLA2B	13.401	0.046
BC042366	12.592	0.016
ADAM29	9.774	0.042
CA9	8.325	0.013
HM587423	8.191	0.043
CLPSL1	7.063	0.021
LCE1E	6.916	0.028
DAPL1	5.343	0.046
THNSL2	5.068	0.012
FAM74A3	0.025	0.035
FAM27B	0.052	0.020
C4orf17	0.062	0.046
BC037850	0.073	0.001
LINC00452	0.075	0.027
LOC100128076	0.081	0.041
OR52W1	0.086	0.010
ASB18	0.089	0.021
MIR3125	0.105	0.009
LEFTY1	0.108	0.033
OR7C1	0.116	0.001
SLC25A52	0.121	0.036
PROKR1	0.138	0.007
DQ786293	0.144	0.023
LOC84931	0.149	0.041
DQ591726	0.149	0.029
OLIG1	0.162	0.040
DQ592690	0.166	0.023
DQ595741	0.179	0.007
BC087858	0.180	0.045
LOC645752	0.182	0.038
AK125237	0.195	0.044
